# Coordinated Responses of Barley in Photosynthesis, Antioxidative System, and Stomatal Regulation Under Flufenoxuron Stress

**DOI:** 10.3390/toxics14090821

**Published:** 2026-09-15

**Authors:** Xia Ning, Yan Zhi, Shuo Wang, Zhaoli Li, Zhihua Ren, Tingting Ku, Guangke Li, Nan Sang

**Affiliations:** Shanxi Key Laboratory of Coal-Based Emerging Pollutant Identification and Risk Control, Research Center of Environment and Health, College of Environment and Resource, Shanxi University, Taiyuan 030006, China; ningxia@sxu.edu.cn (X.N.); zhiyan2@alu.sxu.edu.cn (Y.Z.); wangshuo67@alu.sxu.edu.cn (S.W.); lizhaoli@sxu.edu.cn (Z.L.); zhren@sxu.edu.cn (Z.R.); kutingting@sxu.edu.cn (T.K.); sangnan@sxu.edu.cn (N.S.)

**Keywords:** photosynthetic characteristics, chloroplast ultrastructure, oxidative stress, osmoregulation, phytotoxicity

## Abstract

Flufenoxuron is extensively applied in agricultural pest control; however, its effects on plant physiological processes remain insufficiently understood. Hence, this study evaluated the effects of flufenoxuron on photosynthetic processes and stomatal regulation in barley. The findings indicated that flufenoxuron significantly reduced photosynthetic efficiency and gas-exchange capacity. Furthermore, flufenoxuron exposure markedly increased abscisic acid (ABA) in leaves but not in roots. Nonetheless, reactive oxygen species (ROS) generation and membrane lipid peroxidation were triggered by flufenoxuron. Concomitantly, defense responses were activated, including the upregulation of peroxidase activity and the accumulation of soluble sugars to mitigate osmotic imbalance. Correlation analysis revealed that stomatal conductance (G_s_) showed significant negative correlations with ABA, H_2_O_2_ (hydrogen peroxide), and O_2_·^−^ (superoxide anion) levels. Furthermore, flufenoxuron exposure altered the expression of genes associated with ABA biosynthesis and catabolism, accompanied by changes in the expression of genes related to ABA- and ROS-associated signaling. These findings suggest that ABA- and ROS-associated processes may be involved in stomatal regulation under flufenoxuron exposure. Overall, this study provides a conceptual and mechanistic basis for understanding the phytotoxicity of benzoylurea compounds.

## 1. Introduction

Flufenoxuron (C_21_H_11_ClF_6_N_2_O_3_) is a benzoylurea pesticide, and its mechanism of action specifically targets the synthesis of chitin [1]. It has been widely applied to pest management in forestry and on fruits, vegetables, and other crops across China and South Korea [2,3]. However, flufenoxuron’s structural stability facilitates both environmental persistence and bioaccumulation in lipid-rich tissues [4]. Environmental monitoring studies have detected flufenoxuron in agricultural and environmental matrices, with concentrations of 0.27 ± 0.03 mg/kg in grape residues and up to 18.5 μg/kg in sediments [5,6]. Concurrently, emerging toxicological evidence indicates that flufenoxuron exposure is associated with mitochondrial dysfunction and cardiovascular impairment in non-target organisms [7,8]. Additionally, a case report of human poisoning following oral ingestion of a flufenoxuron-containing insecticide has suggested that this compound may be associated with myocardial injury [9]. Our previous study has demonstrated that flufenoxuron affects the growth and carbohydrate metabolism of barley [10]. The presence of these residues in agricultural and environmental matrices also raises concerns over potential human exposure and health risks.

Photosynthesis is a fundamental physiological process that is highly susceptible to disruption by environmental stressors, including pesticides [11]. Chlorophyll (Chl) and carotenoids (CAR) are key indicators of photosynthetic efficiency, reflecting light absorption and energy conversion capacity. CARs also serve as accessory pigments with vital photoprotective and antioxidant functions [12,13]. The contents and ratios of these pigments (e.g., Chl a/b and Chl/CAR) are sensitive indicators of photosynthetic apparatus functionality. Gao et al. [14] reported that pesticide exposure causes a decline in photosynthetic pigment content over time, thereby directly reducing photosynthetic performance. In addition to their sensitivity to pigments, stomata—the pivotal interfaces via which plants exchange gases (CO_2_ and H_2_O vapor)—are considerably affected by environmental stressors. Stomata are highly sensitive to such stressors, dynamically regulating gas diffusion in response to environmental cues [15]. Stomatal conductance (G_s_) reflects the regulation of gas exchange and is closely associated with transpiration (T_r_), net photosynthetic rate (P_n_), and intercellular CO_2_ concentration (C_i_). Water use efficiency (WUE) is commonly used to evaluate the balance between carbon assimilation and water loss.

Under stress conditions, increased abscisic acid (ABA) levels have been shown to stimulate stomatal closure. This adaptive response enhances stress survival capacity despite concurrent reductions in transpiration and photosynthetic rates under adverse conditions [16,17]. ABA-associated stomatal regulation is closely linked to dynamic changes in reactive oxygen species (ROS) generation and homeostasis. ROS act as important signaling molecules involved in diverse physiological processes, including growth, development, hormone signaling, and stress responses. However, under adverse conditions, ROS production may exceed the capacity of antioxidant defenses, resulting in the accumulation of ROS such as superoxide (O_2_·^−^) and hydrogen peroxide (H_2_O_2_) and the development of oxidative stress [18]. Excessive ROS accumulation can promote membrane lipid peroxidation, damage cellular macromolecules and photosynthetic structures, and consequently impair cellular and physiological functions [19]. Plants counteract ROS accumulation through coordinated enzymatic and nonenzymatic antioxidant systems. For example, excessive ROS accumulation under environmental stress has been associated with impaired chlorophyll biosynthesis and photosynthetic function [20]. To mitigate ROS toxicity, plants deploy a sophisticated antioxidative system comprising both enzymatic and nonenzymatic components. Collectively, these constituents facilitate ROS detoxification by either scavenging them or mitigating their detrimental effects.

Although previous studies have primarily focused on the effects of flufenoxuron on non-target organisms, its phytotoxicity remains poorly understood. In this study, we investigated the phytotoxic responses of barley (*Hordeum vulgare* L.) to flufenoxuron. We focused on photosynthetic performance, oxidative stress, and osmoregulation. Associated physiological and molecular alterations were also examined. By combining physiological, biochemical, ultrastructural, and transcriptional analyses, we further aimed to elucidate the interrelationships among ABA accumulation, ROS burst, and stomatal behavior in barley under flufenoxuron exposure.

## 2. Materials and Methods

### 2.1. Chemicals and Reagents

Flufenoxuron (purity 98.4%, CAS No. 101463-69-8) was supplied by Sigma–Aldrich (Saint Louis, MO, USA). Dimethyl sulfoxide (DMSO, purity > 99.5%, CAS No. 67-68-5) was sourced from Beijing Solarbio Science & Technology Co., Ltd., (Beijing, China). The flufenoxuron stock solution was prepared by dissolving it in DMSO.

### 2.2. Seed Treatment and Experimental Design

The barley seeds were purchased from Hebei Xingnong Fumin Seed Sales Co., Ltd., (Cangzhou, China). Selected plump barley seeds were washed with distilled water and surface sterilized using a 3% H_2_O_2_ solution for 3 min, followed by thorough washing with both tap and distilled water. The seeds were then soaked in distilled water for 4 h and placed in a dark environment at a constant temperature of (25 ± 1) °C. Seeds with root lengths of 1–1.5 cm were selected for subsequent exposure experiments. Afterward, the barley seedlings were grown at (25 ± 1) °C under long-day conditions (14 h of light/10 h of darkness). The experimental groups included a water-only control (Con), a 0.1% DMSO solvent control (Veh), and flufenoxuron treatments at nominal concentrations of 30, 100, and 300 μM. Two-thirds of the exposure solution was replaced daily with freshly prepared corresponding solution at a fixed time. After 7 days of exposure, leaves and roots were collected separately according to the requirements of the subsequent analyses and immediately frozen at −80 °C. For each treatment, three independent biological replicates were established, with 30 barley seedlings included in each replicate.

### 2.3. Measurement of Pigment Content in Barley

Pigments were extracted from 7-day-old barley seedlings. Fresh leaf tissue (0.1 g) was weighed, finely chopped, and homogenized in 9 mL of a 1:1 (*v*/*v*) mixture of 95% ethanol and 80% acetone using a pre-chilled mortar and pestle. The tube was shaken to ensure thorough mixing, and placed in complete darkness at ambient temperature for 24 h with periodic shaking until the leaves turned completely white. Samples were then centrifuged 4000× *g* for 20 min, and collected supernatant for pigment content determination. The concentrations of Chl a, Chl b, and carotenoids (CAR) were determined by measuring absorbance at 663, 645, and 470 nm using a UV-6100S ultraviolet–visible spectrophotometer (Yuanxi Instruments, Shanghai, China), respectively. The concentration of pigments were calculated using the following formula [21]:

$$Chl\;a\;(\mathrm{mg/g})=\frac{(12.7\;\times \;{OD}_{663}-2.69\;\times \;{OD}_{645})\;\times \;V}{1000\;\times \;W}$$

$$Chl\;b\;(\text{mg/g})=\frac{(22.9\;\times \;{OD}_{645}-4.68\;\times {\;OD}_{663})\;\times \;V}{1000\;\times \;W}$$

$$CAR\;(\text{mg/g})=\frac{(1000\;\times \;{OD}_{470}-3.27\;\times \;\mathrm{Chl}\;a-104\;\times \;\mathrm{Chl}\;b)\;\times \;V}{229\;\times \;1000\;\times \;W}$$

where V is the volume of extracting solution, W is the weight of the sample. OD663, OD645, and OD470 represent the absorbance values of the sample measured at 663, 645, and 470 nm, respectively.

### 2.4. Measurement of Gas Exchange

A portable photosynthesis system (CIRAS-3, PP Systems, Amesbury, MA, USA) was used to determine the gaseous exchange parameters of barley leaves after 7 days of exposure at 10:00 a.m.–11:00 a.m.

### 2.5. Transmission Electron Microscopy (TEM) Analyses of Barley Leaves

Three biological replicates per treatment were analyzed. Chlorotic leaf sections were rinsed with ultrapure water, air dried, excised, and immediately fixed in electron microscopy fixative. The samples were sectioned into 1-mm^3^ pieces and transferred to fresh fixative in Eppendorf tubes. After vacuum-assisted fixation, the specimens were stored and transported at 4 °C. Subsequently, the samples were rinsed with 0.1 M phosphate buffer and post-fixed in 1% osmium tetroxide. After additional rinsing in buffer, the samples were subjected to graded dehydration in ethanol and acetone at room temperature. For embedding, the specimens were gradually infiltrated with acetone/812 embedding resin mixtures and ultimately impregnated with pure 812 resin. Polymerization was performed sequentially at 37 °C overnight and at 60 °C for 48 h. Ultrathin sections (70 nm) were prepared using an ultramicrotome and mounted on copper grids. The sections were double-stained with saturated uranyl acetate in 50% ethanol for 15 min, followed by Reynolds’ lead citrate. After final rinsing and drying, the cellular ultrastructure was examined using TEM, and images were systematically captured.

### 2.6. ABA Contents in Barley

Barley leaves and roots were collected, freeze-dried, and ground after harvest. The ABA content in leaves and roots was determined using an enzyme-linked immunosorbent assay kit according to the manufacturer’s instructions (LE-Y1567, LAIER-BIO, Hefei, China). Measurements were conducted in triplicate.

### 2.7. H_2_O_2_, MDA, and O_2_·^−^ Accumulation in Barley

MDA content was measured according to the manufacturer’s instructions using a commercially available kit (Solarbio Science & Technology Co., Ltd., Beijing, China). Briefly, leaf and root samples (0.1 g each) were homogenized in 1 mL of ice-cold extraction buffer. Following centrifugation at 8000× *g* for 10 min at 4 °C, the resultant supernatant was collected and maintained on ice for subsequent analysis.

Histochemical detection of H_2_O_2_ and O_2_·^−^ was performed using DAB and NBT staining kits (Solarbio, Cat. No. G2370 and G4816, respectively), according to the manufacturer’s instructions. Barley leaves were collected, gently rinsed with deionized water, and excess water was blotted onto filter paper. The leaves were then immersed in DAB or NBT staining solution and incubated at room temperature under light-protected conditions for 6 h, until brown (DAB) or dark blue (NBT) precipitates developed in the positive regions while the remaining tissue appeared near colorless or retained its inherent color. To clarify the tissue, the stained leaves were decolorized in 95% ethanol at 40 °C for 12 h to decolorize the chlorophyll. After decolorization, images were captured under identical illumination and exposure conditions. The percentage of stained area was then quantified using ImageJ (version 1.46r) software and expressed as a percentage of the total leaf area analyzed.

### 2.8. Activities of Antioxidant Enzymes

Peroxidase (POD) and superoxide dismutase (SOD) activities were determined using commercial kits (Solarbio Science & Technology Co., Ltd., Beijing, China). Leaf and root samples (0.1 g each) were homogenized in 1 mL ice-cold conditions using a prechilled mortar and pestle. The samples were then centrifuged at 8000× *g* for 10 min at 4 °C, after which the resulting supernatant was collected and stored on ice until further analysis.

### 2.9. Determination of Osmolyte Content

The soluble sugar content was measured using a commercial kit (Solarbio Science & Technology Co., Ltd., Beijing, China), as detailed in Text S1. Soluble protein content was quantified using the Coomassie Brilliant Blue assay [22]. The complete procedure is provided in Text S1.

### 2.10. Quantitative Real-Time Polymerase Chain Reaction (qRT-PCR) Analysis

Detailed procedures for qRT-PCR analysis are provided in Text S2, with reaction systems and amplification parameters summarized in Tables S1–S4. Briefly, total RNA was extracted from barley leaves using TRIzol Reagent (Invitrogen, Carlsbad, CA, USA). Genomic DNA was eliminated by PrimeScript RT reagent kit (Takara, Japan). After reverse transcription, amplification was performed with gene-specific primers and SYBR Green on a qTOWER2.2 real-time PCR system (AnalytikJenaAG, Jena, Germany). The qPCR protocol included initial denaturation at 95 °C for 2 min, followed by 40 cycles of 95 °C for 20 s, 60 °C for 30 s, and 72 °C for 30 s. Relative expression levels were calculated using the 2^−ΔΔCT^ method, normalized to the GAPDH reference gene. Primer sequences are listed in Table S5 [23,24].

### 2.11. Data Analysis

The results are presented as mean ± standard deviation (SD) based on three independent biological replicates. Each independent biological replicate was treated as the experimental unit for statistical analysis. Statistical analyses were performed using GraphPad Prism 8.0. Differences among treatment groups were evaluated using one-way analysis of variance (one-way ANOVA), followed by Dunnett’s multiple-comparisons test with the Veh group as the reference. Statistical significance was set at *p* < 0.05. Graphs were generated using GraphPad Prism 8.0 and R version 4.2.

## 3. Results

### 3.1. Measurements of Photosynthetic Pigments and Gas Exchange in Barley

**Figure 1:**
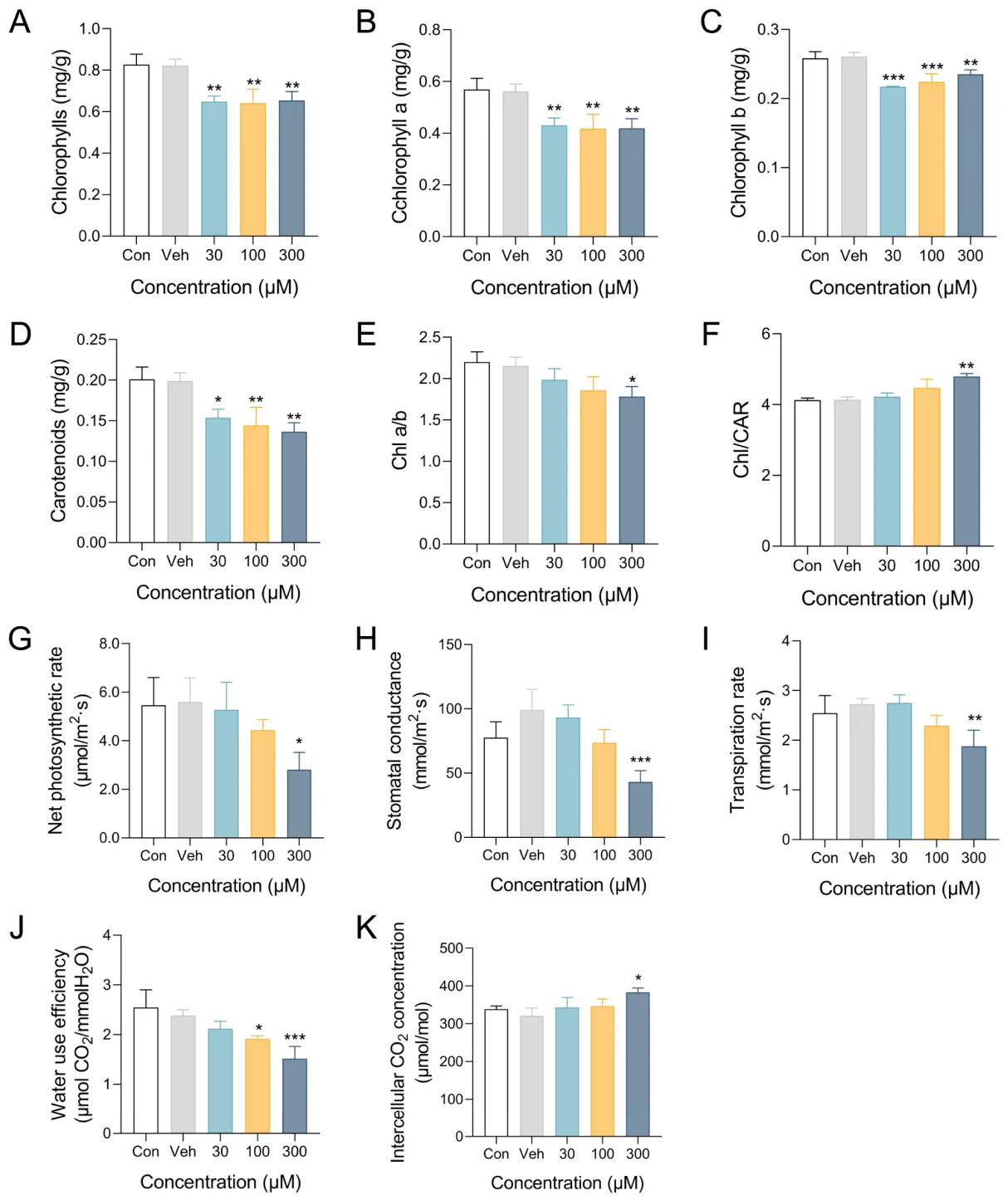
The effect of flufenoxuron on the photosynthetic pigments (**A**–**F**) and the photosynthetic parameters P_n_ (**G**), G_s_ (**H**), T_r_ (**I**), WUE (**J**), and C_i_ (**K**) of barley. Data are presented as mean ± SD (*n* = 3). Statistical significance was determined using one-way ANOVA followed by Dunnett’s multiple-comparisons test. Significance for each data point is denoted by asterisks (* *p* < 0.05, ** *p* < 0.01, and *** *p* < 0.001). Chl, chlorophylls; Chl a, chlorophyll a; Chl b, chlorophyll b; CAR, carotenoids; P_n_, net photosynthetic rate; G_s_, stomatal conductance; T_r_, transpiration rate; WUE, water use efficiency. C_i_, intercellular carbon dioxide concentration.

This study investigated the effects of flufenoxuron on photosynthetic pigment content and gas-exchange parameters in barley seedlings. As depicted in Figure 1A, compared with the Veh group, the total Chl content was significantly reduced (20.45%) in the 300 μM treatment group. Furthermore, the contents of Chl a, Chl b, and CAR fell drastically by 25.34%, 9.91%, and 31.26% (Figure 1B–D), respectively. Concurrently, flufenoxuron treatment decreased the Chl a/b (Figure 1E) ratio but increased the Chl/CAR ratio (Figure 1F). Notably, P_n_ (49.70%), G_s_ (56.43%), T_r_ (31.11%), and WUE (36.37%) were significantly reduced in the 300 μM treatment group (Figure 1G–J). By contrast, C_i_ exhibited a significant increase of 19.31% (Figure 1K). In summary, flufenoxuron treatment significantly lowered the contents of photosynthetic pigments and gas-exchange measurements, potentially affecting plant photosynthesis.

### 3.2. The Ultrastructure of Chloroplasts in Barley

**Figure 2:**
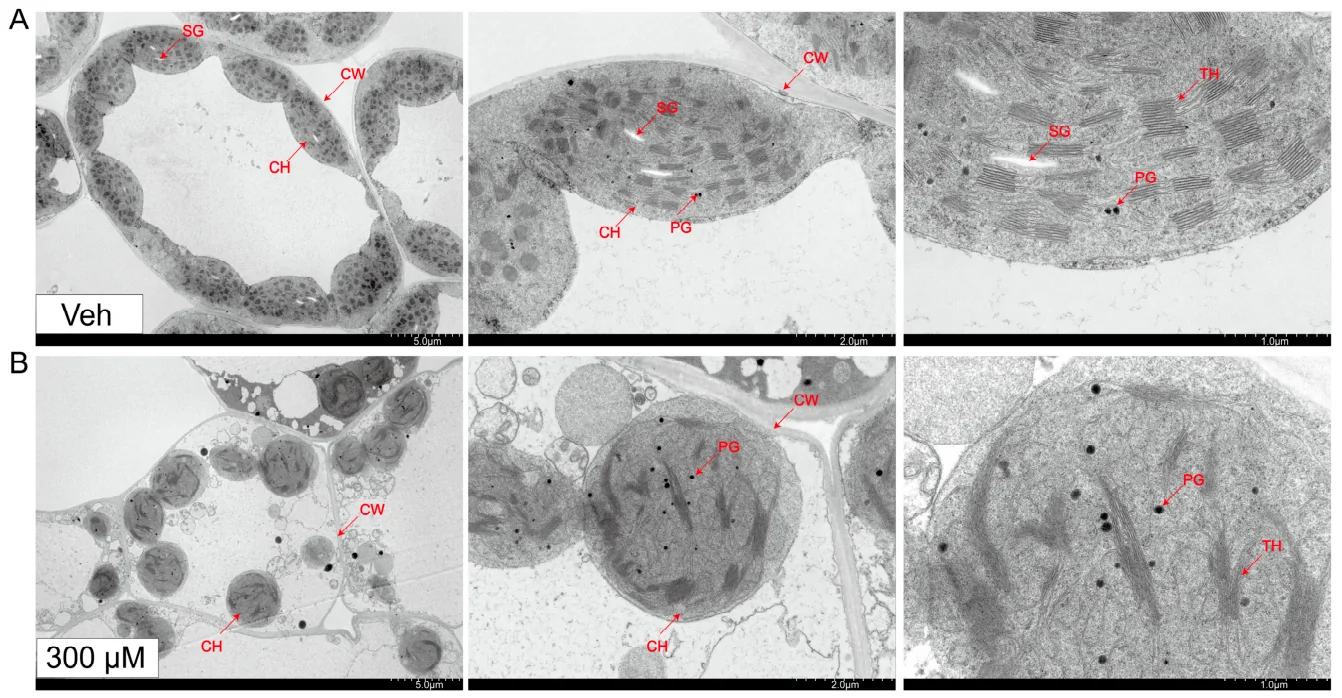
Transmission electron micrographs of chloroplasts from barley plants grown on different flufenoxuron concentrations. (**A**) Chloroplasts of barley in the Veh. (**B**) Chloroplasts of barley grown on 300 μM flufenoxuron. CH, chloroplast; CW, cell wall; SG, starch granule; PG, plastoglobuli; TH, thylakoid.

TEM revealed marked ultrastructural alterations in the chloroplasts of flufenoxuron-treated barley leaves (Figure 2). Chloroplasts in Veh group exhibited intact, well-organized morphology with clearly defined membranes, prominent starch granules, and uniformly stacked thylakoids (Figure 2A). In contrast to the Veh group, chloroplasts in the FF-treated group changed from their normal spindle shape to a swollen, spherical morphology (Figure 2B). The grana lamellae became loosened, and the thylakoid arrangement was disorganized. In addition, starch granules almost completely disappeared, while the number and the size of plastoglobuli were enlarged. These changes indicate enhanced lipid peroxidation and oxidative damage to the membrane system. Notably, a marked proliferation of plastoglobuli was observed, accompanied by a concurrent increase in their individual volume.

### 3.3. ABA Content in Barley

**Figure 3:**
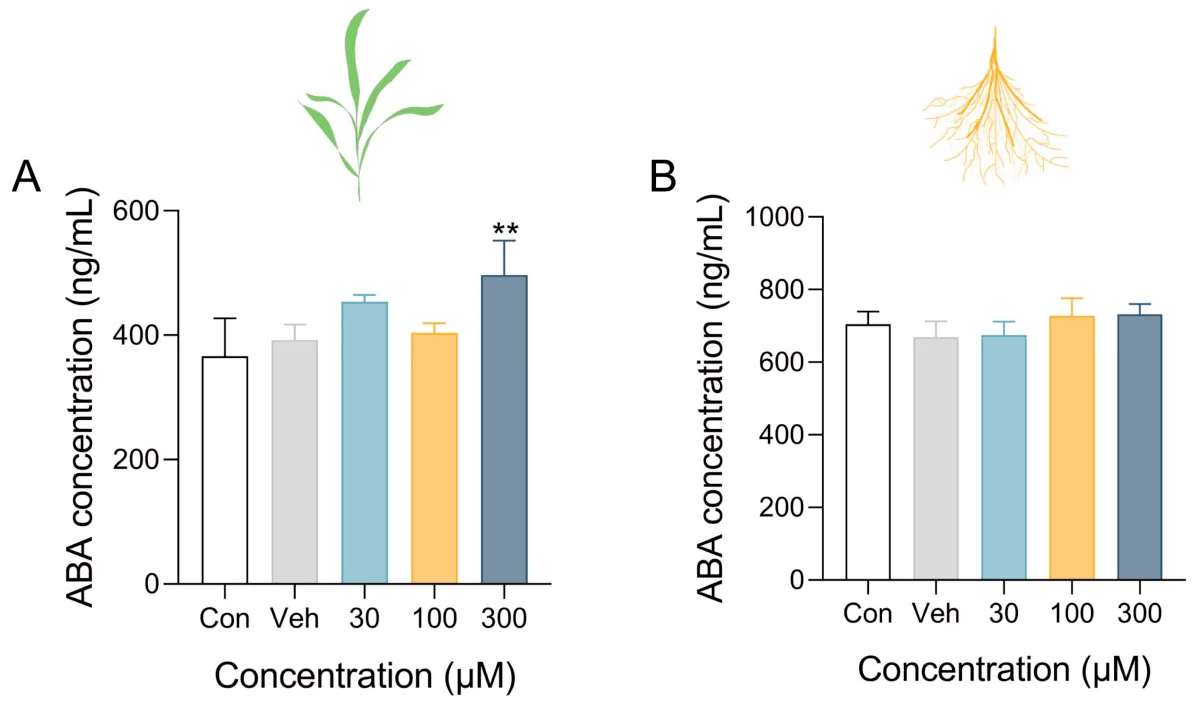
The effects of flufenoxuron on ABA content. (**A**) The content of ABA in the leaves. (**B**) The content of ABA in the roots. Data are presented as mean ± SD (*n* = 3). Statistical significance was determined using one-way ANOVA followed by Dunnett’s multiple-comparisons test. Significance for each data point is denoted by asterisks (** *p* < 0.01).

The results demonstrated that the distribution characteristics of ABA were significantly altered in the 300 μM treatment group. In the leaf tissue, a significant 26.70% increase in ABA was induced in the 300 μM treatment group compared with the Veh group (Figure 3A). Conversely, no significant alterations were observed in root tissues in any of the treatment groups (Figure 3B).

### 3.4. ROS Content and Membrane Lipid Peroxidation

**Figure 4:**
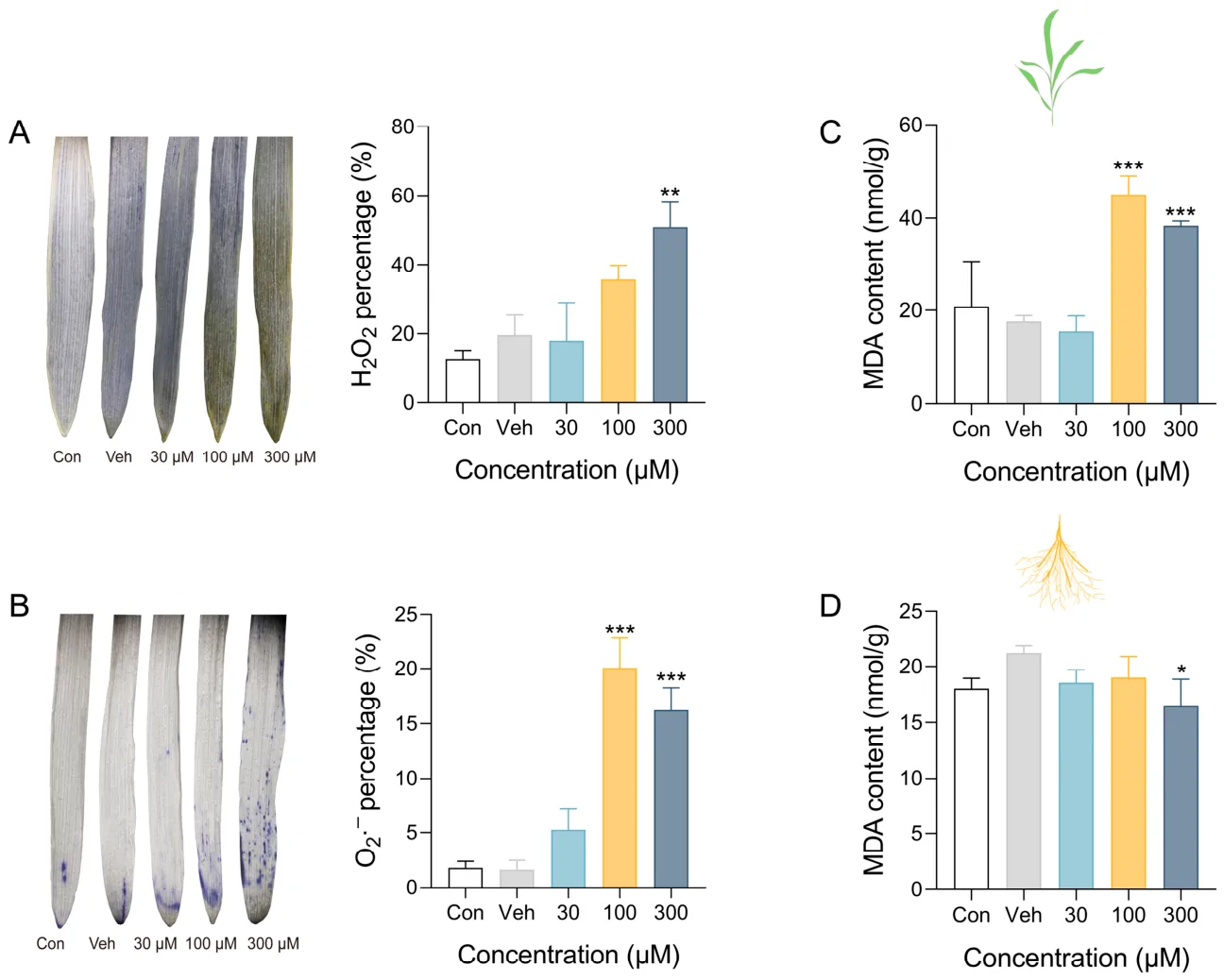
The effects of flufenoxuron treatments on ROS accumulation and membrane lipid peroxidation, visualized in barley leaves and roots. (**A**) Accumulation of H_2_O_2_ detected with DAB staining. (**B**) Accumulation of O_2_·^−^ detected with NBT staining. (**C**) Leaf MDA content. (**D**) Root MDA content. Data are presented as mean ± SD (*n* = 3). Statistical significance was determined using one-way ANOVA followed by Dunnett’s multiple-comparisons test. Significance for each data point is denoted by asterisks (* *p* < 0.05, ** *p* < 0.01, and *** *p* < 0.001). H_2_O_2_, hydrogen peroxide; O_2_·^−^, superoxide anion; MDA, malondialdehyde.

Oxidative stress levels were evaluated in barley exposed to flufenoxuron. The findings revealed pronounced oxidative damage in leaves in the 300 μM treatment group, with H_2_O_2_ and O_2_·^−^ levels elevated to 260.39% and 1004.99% of the Veh group values, respectively (Figure 4A,B). Similarly, the MDA content in the leaves was considerably upregulated as the concentration of flufenoxuron increased (Figure 4C,D). At concentrations of 100 μM and 300 μM, MDA accumulation increased sharply by 154.62% and 117.49%, respectively, compared with the Veh group. Contrastingly, root MDA content displayed an inverse trend, decreasing significantly by 21.96% at 300 μM. Collectively, these results indicated severe flufenoxuron-induced oxidative stress in leaves, characterized by ROS accumulation and lipid peroxidation.

### 3.5. Antioxidant Enzyme Activities in Barley

**Figure 5:**
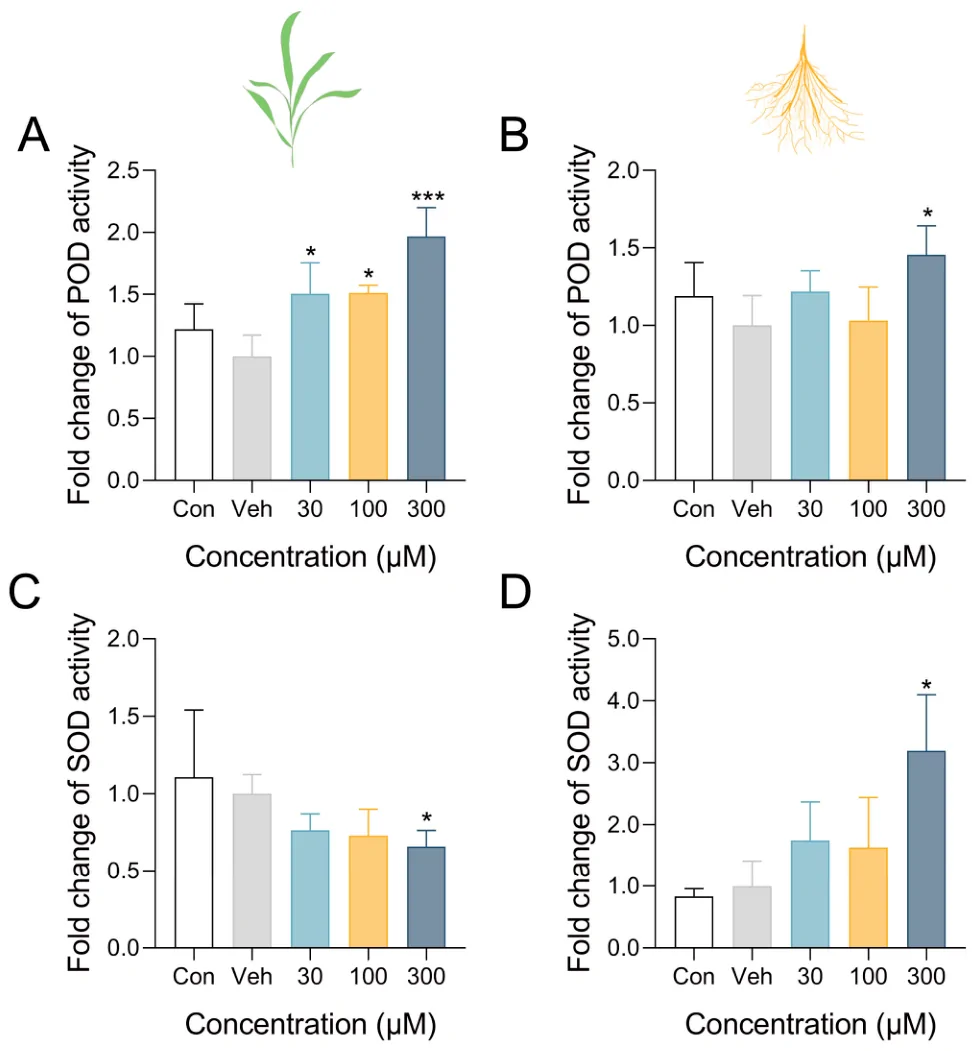
The effects of flufenoxuron treatments on the levels of antioxidant enzyme activities in barley. POD activity in leaves (**A**) and roots (**B**), and SOD activity in leaves (**C**) and roots (**D**). Data are presented as mean ± SD (*n* = 3). Statistical significance was determined using one-way ANOVA followed by Dunnett’s multiple-comparisons test. Significance for each data point is denoted by asterisks (* *p* < 0.05, and *** *p* < 0.001). POD, peroxidase; SOD, superoxide dismutase.

Flufenoxuron exposure triggered substantial oxidative stress in barley, activating tissue-specific antioxidant responses. Specifically, in the 300 μM treatment group, POD activity increased significantly by 96.77% in leaves and 45.53% in roots (Figure 5A,B). Unlike the alterations in POD activity, SOD displayed a tissue-specific regulatory pattern. In the leaf tissue, SOD activity was significantly downregulated by 34.15% in the 300 μM treatment group (Figure 5C). However, SOD was markedly activated in the roots, with its relative activity increasing to 319.32% of that in the Veh group (Figure 5D).

### 3.6. Osmotic Regulation in Barley

**Figure 6:**
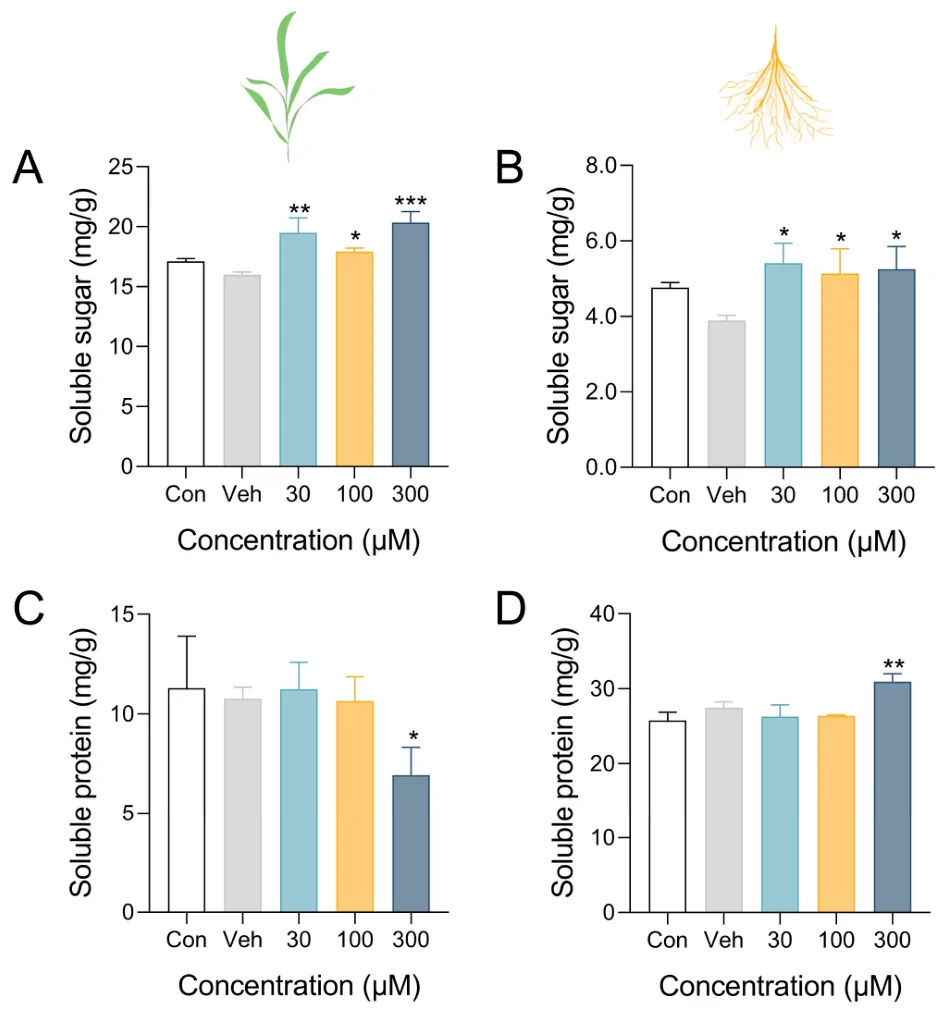
The effects of flufenoxuron treatments on osmotic regulation in barley. (**A**) Soluble sugar content in leaves. (**B**) Soluble sugar content in roots. (**C**) Soluble protein content in leaves. (**D**) Soluble protein content in roots. Data are presented as mean ± SD (*n* = 3). Statistical significance was determined using one-way ANOVA followed by Dunnett’s multiple-comparisons test. Significance for each data point is denoted by asterisks (* *p* < 0.05, ** *p* < 0.01, and *** *p* < 0.001).

Changes in soluble sugar and soluble protein content reflect barley’s osmotic regulation capacity. Compared with the Veh group, the soluble sugar content was increased significantly by 27.22% in leaves and 34.99% in roots (Figure 6A,B). Nonetheless, soluble protein content in the leaves decreased significantly by 35.62%, whereas in the roots it increased slightly by 12.64% in the 300 μM treatment group (Figure 6C,D).

### 3.7. ABA and ROS Mediated the Stomatal Response

**Figure 7:**
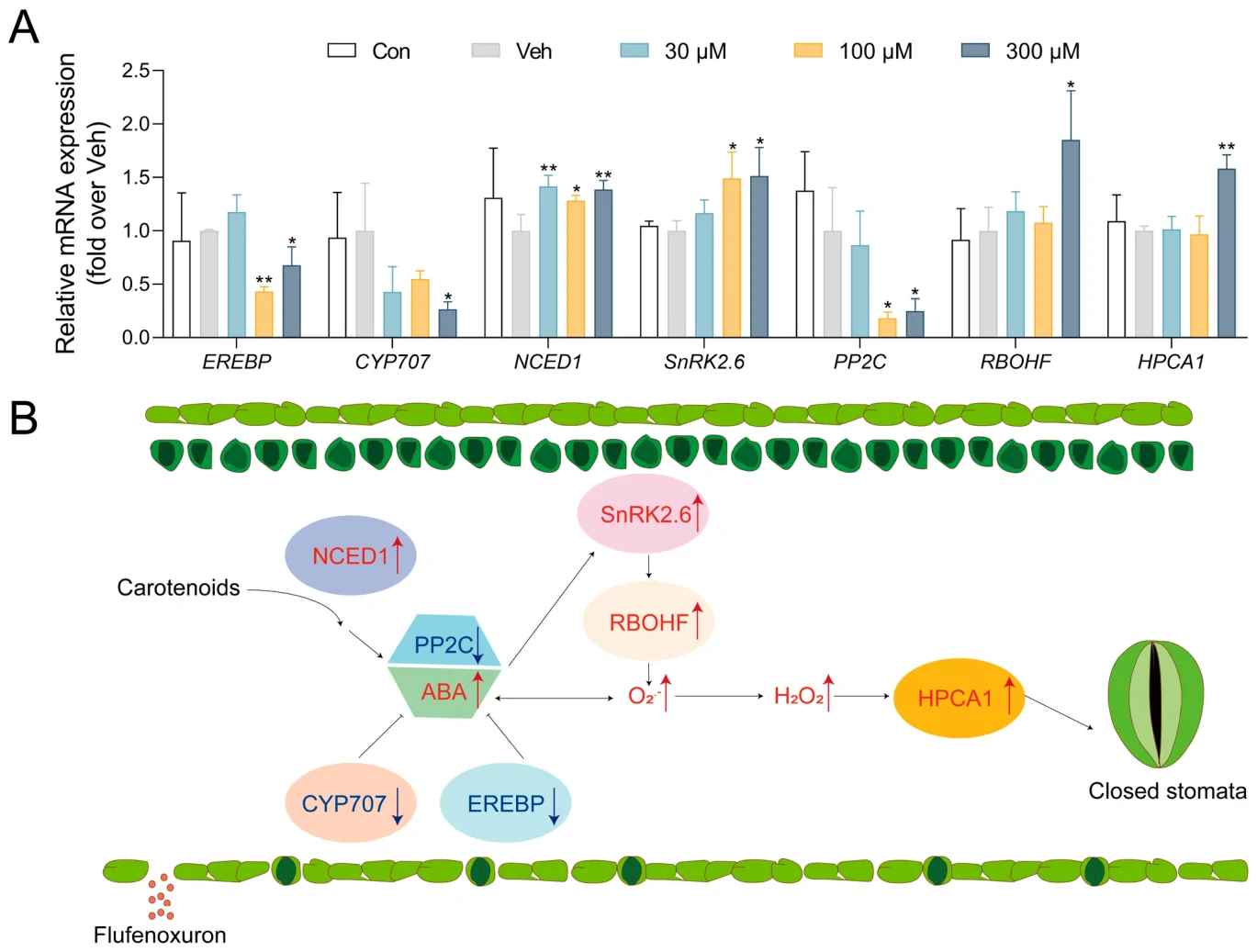
Stomatal regulation pathways mediated by genes. (**A**) Analysis of the expression of genes involved in the stomatal regulation pathway. (**B**) Schematic of the stomatal regulation pathway regulated by genes. Data are presented as mean ± SD (*n* = 3). Statistical significance was determined using one-way ANOVA followed by Dunnett’s multiple-comparisons test. Significance for each data point is denoted by asterisks (* *p* < 0.05, and ** *p* < 0.01). Increases and decreases are represented by red and blue colors, respectively.

Correlational analysis of various indicators demonstrated that G_s_ was significantly negatively correlated with ABA, H_2_O_2_, and O_2_·^−^ (Figure S1). These associations suggest that ABA- and ROS-associated responses may be involved in the regulation of stomatal conductance under flufenoxuron exposure. The expression levels of *EREBP* and *CYP707* were significantly downregulated in the 300 μM treatment group, reaching 67.77% and 26.47% of the Veh group levels, respectively (Figure 7A). Conversely, the expression levels of *NCED1* and *SnRK2.6* were markedly upregulated under flufenoxuron treatment. The expression level of *PP2C* was significantly suppressed by flufenoxuron treatment. By contrast, the expression levels of *RBOHF* and *HPCA1* were significantly upregulated following flufenoxuron exposure. A proposed model illustrating the potential involvement of ABA- and ROS-associated processes in stomatal regulation under flufenoxuron exposure is presented in Figure 7B.

## 4. Discussion

It is well established that insecticides pose substantial ecophysiological risks to crop plants, including photosynthetic inhibition, oxidative damage, osmotic imbalance, and hormonal disruption [25]. The results revealed that flufenoxuron significantly reduced the photosynthetic pigment content in barley leaves. This finding aligns with prior reports of pesticide-induced photosynthetic inhibition [26]. Chlorophyll content directly influences photosynthetic capacity [27]. The Chl a/b ratio has been widely used as an indicator of the structural and functional status of the photosynthetic apparatus; therefore, the observed 17.2% decline in the Chl a/b ratio may be associated with alterations in PSII structure or function [28]. Such changes may affect light-harvesting and photoprotective functions, consistent with the observed ultrastructural damage to thylakoids. In photosynthesis, CAR functions as an antioxidant, scavenging ROS and inhibiting lipid peroxidation [29]. Moreover, CAR acts as a precursor for ABA biosynthesis, a critical regulator of stress-responsive stomatal closure [30].

Photosynthetic efficiency is governed by pigment content coupled with gas-exchange dynamics. Classical theory suggests that under flufenoxuron stress, ABA-mediated stomatal regulation (reduced G_s_) minimizes water loss (decreased T_r_) while restricting CO_2_ diffusion (lowered C_i_), ultimately reducing P_n_ [31,32]. On the contrary, an atypical response was observed in this study: concomitant reductions in G_s_ and P_n_ were accompanied by significantly elevated C_i_. This pattern suggests that the reduction in photosynthetic performance may involve substantial non-stomatal limitations in addition to changes in stomatal regulation. The observed reductions in photosynthetic pigments and alterations in chloroplast ultrastructure may be associated with the observed impairment of photosynthetic performance [33].

The ultrastructure of chloroplasts indicated that flufenoxuron stress altered the ultrastructure of leaf mesophyll cells, with an increase in the size and number of plastoglobuli and disorganization of the thylakoid arrangement. An increase in plastoglobuli has been reported in plants exposed to various environmental stresses and may be associated with changes in chloroplast membrane lipid metabolism [34]. The observed chloroplast alterations, together with the changes in photosynthetic pigments and ROS-related parameters, may be associated with the impaired photosynthetic performance observed under flufenoxuron exposure.

As a central stress regulator, ABA modulates seed dormancy, stomatal closure, and stress adaptation [35,36]. In this study, flufenoxuron induced a significant accumulation of ABA, consistent with herbicide-induced ABA elevation [37]. Beyond serving as a stress biomarker, ABA functions as a signaling hub that can initiate downstream stress responses. Importantly, ROS play a dual role in this process. At moderate levels, ROS act as signaling molecules that can participate in ABA-induced stomatal closure and stress adaptation. However, when ROS production exceeds the cellular scavenging capacity, their accumulation becomes detrimental. Under flufenoxuron stress, excessive ROS may disrupt mitochondrial electron transport and enhance lipid peroxidation, thereby compromising membrane integrity [38]. This shift from ROS-mediated signaling to oxidative toxicity may contribute to the impairment of the photosynthetic system under flufenoxuron stress.

Plants have evolved complex antioxidant systems to counteract ROS and maintain redox homeostasis. Under flufenoxuron stress, barley exhibited distinct enzyme-specific responses. The significant increase in leaf POD activity and the significant decrease in SOD activity indicated that barley leaves were subjected to oxidative stress [39]. In the plant oxidative stress response system, dynamic alterations in POD activity are considered sensitive indicators of stress [40]. The increase in POD activity may represent a compensatory defense mechanism to scavenge excessive H_2_O_2_. However, POD can also contribute to catalyzed Chl degradation [41], potentially explaining the observed reduction in photosynthetic pigments. Conversely, significantly reduced SOD activity may weaken the capacity to scavenge O_2_·^−^, potentially leading to its accumulation and exacerbating oxidative stress. Contrastingly, roots displayed considerably augmented SOD activity, implying a potentially enhanced antioxidant response in subterranean organs [40].

Flufenoxuron altered cellular osmotic homeostasis in barley. Our observations revealed a consistent increase in soluble sugar content in both roots and leaves under flufenoxuron stress. This finding suggests that barley may accumulate soluble sugars as part of an osmotic adjustment response, which could help maintain turgor pressure and facilitate water uptake, thereby alleviating osmotic stress. Conversely, the SP content showed an opposite trend, with a decrease in leaf protein content under flufenoxuron stress. This observation could be explained by accelerated protein degradation under stress, leading to decreased soluble protein content. By contrast, the soluble protein content in roots showed an increasing trend. This tissue-specific response may be associated with differential physiological and metabolic responses between roots and leaves under flufenoxuron exposure [42].

Correlation analysis revealed that G_s_ was significantly negatively correlated with ABA, H_2_O_2_, and O_2_·^−^ levels, suggesting an association between stomatal regulation and ABA- and ROS-related responses under flufenoxuron exposure. The results showed that flufenoxuron treatment significantly upregulated the ABA biosynthetic gene *NCED1*, while downregulating the catabolism-related genes *EREBP* and *CYP707*, which was accompanied by increased ABA accumulation in leaves. In addition, *PP2C* expression was downregulated, whereas *SnRK2.6* expression was upregulated, suggesting a possible alteration of ABA signaling under flufenoxuron exposure [43,44]. These changes were accompanied by increased ROS accumulation and upregulation of *RBOHF* expression, which is consistent with a potential role of *RBOHF* in ROS production [45,46]. Increased H_2_O_2_ levels may also be associated with *HPCA1*-mediated ROS sensing and downstream Ca^2+^ signaling, which have been implicated in stomatal regulation in previous studies [47,48]. Collectively, our results suggest that alterations in ABA accumulation, ROS levels, and the expression of ABA- and ROS-related genes may be involved in stomatal regulation under flufenoxuron exposure. Based on the current understanding of ABA- and ROS-related signaling and the molecular responses observed in this study, we propose a model illustrating the potential involvement of ABA- and ROS-associated processes in stomatal regulation under flufenoxuron exposure.

Several limitations require emphasis. First, this study employed a short-term seedling exposure system, focusing primarily on photosynthesis, antioxidant systems, hormone metabolism, and stomatal regulation. As such, it does not fully capture the potential effects of long-term, low-dose, or environmentally variable exposure on barley. Second, although ABA–ROS involvement is suggested by gas exchange and gene expression data, we did not measure stomatal aperture or apply genetic/pharmacological validation, so this mechanism remains a working model. Therefore, the proposed mechanism should be regarded as a working model based on multi-level evidence, rather than a directly validated causal mechanism. Despite these constraints, our findings establish a foundational mechanistic framework. Future studies should extend the exposure regime to long-term, low-dose, and environmentally realistic scenarios to better assess ecological risks. Future studies should combine environmentally realistic long-term low-dose exposure, stomatal observations, genetic and pharmacological interventions, and cross-species multi-omics approaches to conclusively confirm the ABA-ROS axis and improve non-target plant ecological risk assessment.

## 5. Conclusions

The findings from this study showed that flufenoxuron significantly increased ABA levels in barley leaves. Concurrently, pigment content decreased, and photosynthetic parameters were substantially altered. In addition, flufenoxuron application induced severe oxidative stress, as evidenced by considerable increases in H_2_O_2_, O_2_·^−^, and MDA levels. These changes were accompanied by ultrastructural damage to chloroplasts, which may have contributed to impaired photosynthetic function. At the molecular level, flufenoxuron exposure altered the expression of genes associated with ABA biosynthesis, ABA catabolism, and ROS-related signaling, suggesting a possible involvement of ABA- and ROS-associated processes in stomatal regulation. Overall, this study provides new insights into the coordinated physiological responses of barley to flufenoxuron exposure, particularly in relation to photosynthesis, oxidative stress, and stomatal regulation.

## Data Availability

The data supporting the findings of this study are available from the authors upon reasonable request.

## Supplementary Materials

The following supporting information can be downloaded at https://www.mdpi.com/article/10.3390/toxics14090821/s1; Figure S1: Correlation analysis among various indicators; Table S1: Genomic DNA removal reaction system. Table S2: Reverse transcription reaction system. Table S3: PCR reaction system. Table S4: PCR reaction procedure. Table S5: Primer Sequences; Text S1: Determination of osmolyte content; Text S2: Quantitative real-time polymerase chain reaction (qRT-PCR) analysis.
