# Supplementary material for: Coordinated Responses of Barley in Photosynthesis, Antioxidative System, and Stomatal Regulation Under Flufenoxuron Stress
Source: Toxics. 2026 Sep 15;14(9):821. doi: 10.3390/toxics14090821 (PMC13611293; doi:10.3390/toxics14090821)
Supplement: Supplementary file 1 [file toxics-14-00821-s001.zip › toxics-4468167-supplementary.pdf]

Coordinated Responses of Barley in Photosynthesis, Antioxidative System, and Stomatal Regulation under Flufenoxuron Stress

Contents of Supplementary Materials

Manuscript Title	Coordinated Responses of Barley in Photosynthesis, Antioxidative System, and Stomatal Regulation under Flufenoxuron Stress
Complete List of Authors	Xia Ning, Yan Zhi, Shuo Wang, Zhaoli Li, Zhihua Ren, Tingting Ku, Guangke Li, Nan Sang
Total Pages	9
Total Texts	2
Total Tables	5
Total Figures	1

Text S1. Determination of osmolyte content

The content of soluble sugar (SS) was determined using a commercial kit (Solarbio Science & Technology Co., Ltd., Beijing, China). Barley tissue exposed for 7 days was collected, ground to a fine powder in liquid nitrogen, and approximately 0.2 g of the sample was homogenized in 1 mL of distilled water. The extraction process entailed an initial 10-minute incubation in a boiling water bath, followed by cooling and centrifugation (8000 × g, 10 min, room temperature). The final volume of the mixture was adjusted to 10 mL with distilled water, and the solution was thoroughly agitated prior to analysis.

For soluble protein measurement, approximately 500 mg of fresh barley leaves was homogenized thoroughly in 2 mL of distilled water using a mortar and pestle until a uniform homogenate was obtained. The homogenate was transferred to a centrifuge tube and allowed to stand at room temperature for 50 min, followed by centrifugation at 4000 rpm for 20 min. The resulting supernatant was collected and transferred to a volumetric flask, and distilled water was added to a final volume of 10 mL to obtain the sample extract. Subsequently, 0.1 mL of the sample extract was transferred into each of two test tubes, followed by the addition of 5 mL of Coomassie Brilliant Blue G-250 reagent. The solutions were thoroughly mixed and allowed to react for 60 s. The absorbance was then measured at 595 nm using a spectrophotometer, and the readings were recorded.

$$\text{SP content (mg/g)} = \frac{X \times V_T \times N}{W \times V_s \times 1000}$$

Where:

X - Protein mass determined from the standard curve (μg);

V_T - Total volume of the extraction solution (mL);

N - Dilution factor;

W - Barley mass (g);

V_S - Volume of sample solution used for measurement (mL).

Text S2. Quantitative real-time polymerase chain reaction (qRT-PCR) analysis

1. RNA extraction

Total RNA was extracted from barley leaf samples using TRIzol Reagent. Briefly, approximately 0.1 g of frozen leaf tissue was rapidly ground in liquid nitrogen and homogenized in 1 mL of TRIzol Reagent. After incubation at room temperature for 5 min, 0.2 mL of chloroform was added, followed by vigorous shaking for 30 s and incubation on ice for 5 min. The samples were centrifuged at 10,000 rpm for 10 min at 4°C, and the upper aqueous phase was collected. Subsequently, 0.2 mL of chloroform was added, followed by vigorous shaking for 30 s and incubation on ice for 5 min. The samples were centrifuged at 13,500 rpm for 10 min at 4°C, and the upper aqueous phase was collected. RNA was precipitated by adding 0.5 mL of isopropanol, followed by gentle inversion and incubation on ice for 5 min. The samples were then centrifuged at 13,500 rpm for 10 min at 4°C, and the supernatant was discarded. The RNA pellet was washed with 1 mL of 75% ethanol and centrifuged at 13,500 rpm for 10 min at 4°C. The pellet was air-dried at room temperature and dissolved in approximately 20 μL of DEPC-treated water. RNA concentration was subsequently determined.

2. Reverse transcription of RNA into cDNA

First-strand cDNA was synthesized using the PrimeScript RT reagent Kit (TaKaRa, Japan) according to the manufacturer's instructions.

a. Genomic DNA elimination: The reaction mixture was prepared as listed in Table S1 and incubated at 42 °C for 2 min, followed by 5 min at 4 °C.

Table S1 Genomic DNA removal reaction system

Component	Volume
gDNA Eraser	1 μL
5×gDNA Eraser Buffer	2 μL
RNA	7 μL

b. The reverse transcription reaction was performed in a system prepared as described in Table S2.

Table S2 Reverse transcription reaction system

Component	Volume
PrimerScript RT Enzyme Mix I	1 μL
5×PrimerScript Buffer 2	4 μL
RT Primer Mix	1 μL
RNase Free dH_2O	4 μL
The above reaction solution from step a	10 μL

3. PCR amplification

PCR was performed using the reaction system and thermal cycling conditions described in Table S3 and Table S4, respectively.

Table S3 PCR reaction system

Component	Volume
2×M5 HiPer Realtime PCR Super Mix	10 μL
Primer F	1 μL
Primer R	1 μL
RNase free water	6 μL
cDNA	2 μL

Table S4 PCR reaction procedure

Component	Volume
95℃	2 min
95℃	20 sec
60℃	30 sec
72℃	30 sec

Table S5 Primer Sequences

Gene	Database identifier	Forward primer (5'→3')	Reverse primer (5'→3')	Source
GAPDH	HvGAPDH	ACCACTAACTGCCTTGCTCC	ATGCTGGACCTGTTGTCACC	NCBI GenBank
SnRK2.6	HORVU0Hr1G011570	ATGTGTGGTCTTGTGGGGTG	GATGACTTCGGCATGGTCCT	Ensemble Plants
CYP707	HvABA8'OH-1	CTCAAAGCCGTCACCGAAGA	CCACCTCGAATCGTGAAGGA	[23]
EREBP	AAM91511.1	CGAAGCAGAGTGTTGGAAGTACC	AGTAAGTGAAGAAACGGCGATAGTG	NCBI GenBank
RBOHF	HORVU.MOREX.r3.6HG0576260.1	TCCCACTTAGCCACAACGAC	CTCGACGCTACGAAGAACGA	Ensemble Plants
NCED1	HvNCED1	CCAGCACTAATCGATTCC	GAGAGTGGTGATGAGTAA	[23]
HPCA1	HORVU.MOREX.r3.6HG0548430	AACACCCAAGGCATCACCAA	TCCTTGTAGATGAGCCCCGA	Ensemble Plants
PP2C	HvPP2C	CCATCCTTAGAGCAGAGCCG	GAGGTACTGCGACTGACGAG	[24]

23. Seiler, C.; Harshavardhan, V.T.; Rajesh, K.; Reddy, P.S.; Strickert, M.; Rolletschek, H.; Scholz, U.; Wobus, U.; Sreenivasulu, N. ABA biosynthesis and degradation contributing to ABA homeostasis during barley seed development under control and terminal drought-stress conditions. *J. Exp. Bot*. **2022**, *73*, 2615-2632.

24. Wu, X.T.; Xiong, Z.P.; Chen, K.X.; Zhao, G.R.; Feng, K.R.; Li, X.H.; Li, X.R.; Tian, Z.; Huo, F.L.; Wang, M.X.; Song, W. Genome-Wide Identification and Transcriptional Expression Profiles of PP2C in the Barley (*Hordeum vulgare L.*) Pan-Genome. *Genes.* **2022**, *13*, 834.

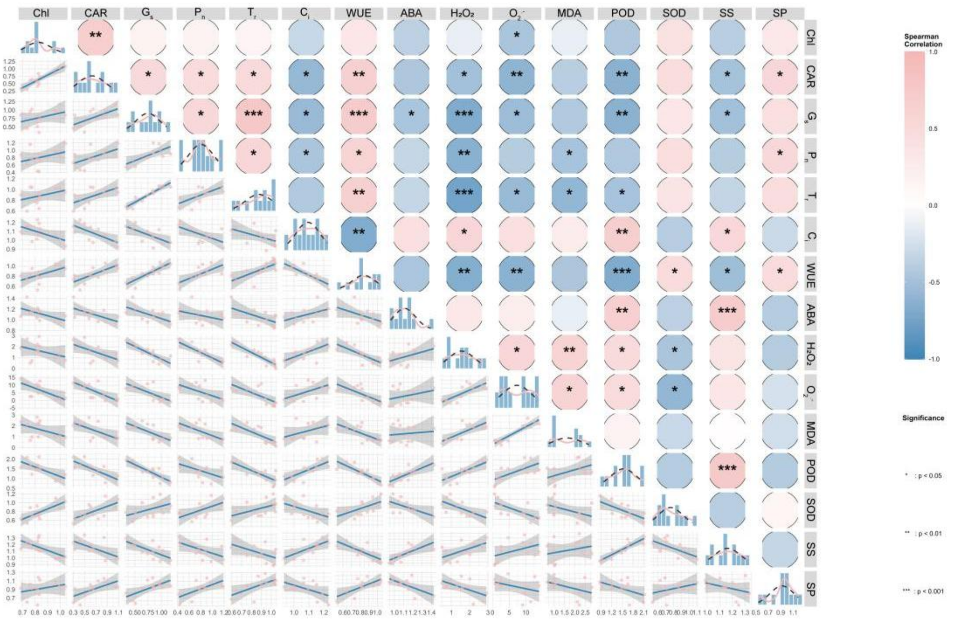

Figure S1. Correlation analysis among various indicators.
